# Within‐Host Evolution of Foot‐and‐Mouth Disease Virus From Persistently Infected Cattle and Buffaloes in Vietnam

**DOI:** 10.1155/tbed/3373628

**Published:** 2026-10-23

**Authors:** Dennis N. Makau, J. Trevor Vannatta, Miranda Bertram, Ian Fish, Jonathan Arzt, Kimberly VanderWaal

**Affiliations:** ^1^ Department of Biomedical and Diagnostic Sciences University of Tennessee Knoxville Knoxville 37996 Tennessee USA utk.edu; ^2^ Department of Biological Sciences Minnesota State University Mankato 56001 Minnesota USA mnsu.edu; ^3^ Foreign Animal Disease Research Unit Agricultural Research Service United States Department of Agriculture Plum Island Animal Disease Center National Bio and Agro-Defense Facility Orient Pt 11957 New York USA usda.gov; ^4^ Department of Veterinary Population Medicine University of Minnesota St. Paul 55108 Minnesota USA umn.edu

## Abstract

Foot‐and‐mouth disease virus (FMDV) persists endemically through carrier animals that maintain infection >21 days postdetection. We characterized within‐host FMDV evolution in persistently infected cattle (*n* = 168) and buffalo (*n* = 16) from northern Vietnam using longitudinal oropharyngeal sampling (3–4 month intervals) under non‐outbreak conditions. Animals remained rRT‐PCR positive without clinical FMD. Persistent infections were defined by <2% VP1 nucleotide divergence across sampling points; serial infections were identified by >5% divergence between sequential samples. From 120 sequences across 38 persistently infected hosts (PIHs), 13.3% of apparent carriers (rRT‐PCR positive >21 days) were actually serially infected with distinct strains. This emphasizes the need for sequence confirmation alongside OPF positivity when estimating carrier prevalence in high‐diversity settings. True persistent infections (*n* = 33 animals) showed rapid VP1 evolution (0.081 mutations/nt site/yr), with diversifying selection strongest in VP1 and VP3 capsid regions. Serotype O PIH exhibited higher VP3 mutation rates than serotype A. Interserotypic recombination was also detected, consistent with carrier animals serving as viral mixing vessels. These field data confirm high evolutionary rates and recombination within FMDV carriers under natural conditions, extending experimental superinfection findings to endemic systems. Results support targeted PIH detection and management to disrupt FMDV persistence and evolution in resource‐limited regions.

## 1. Introduction

While epidemiological theory predicts that highly contagious pathogens are expected to go extinct in populations following an outbreak, the presence of persistently infected hosts (PIHs) (also referred to as carriers) creates an avenue for the pathogen to avoid extinction [1–3]. PIHs may serve as reservoirs, harboring the pathogen without clinical disease and allowing it to survive periods when there is a lack of susceptible hosts. The presence of PIHs may lead to sporadic outbreaks when the conditions are favorable. Persistent infections are not isolated phenomena but reflect a broader ecological pattern where chronic carriers facilitate the survival and spread of pathogens across different species and ecosystems [4]. For instance, an African buffalo (*Syncerus caffer*) can remain persistently infected by foot‐and‐mouth disease virus (FMDV) (*Aphthovirus vesiculae*) for up to 5 years, demonstrating their role as efficient long‐term carriers and the potential for PIHs to spawn outbreaks in susceptible populations, such as domestic cattle [4–7]. Domestic cattle also have been demonstrated to harbor persistent infections (for at least up to 28 months), but natural transmission from a persistently infected cow has never been demonstrated [1, 8]. While the existence of persistently infected cattle guides FMDV control policies, the role of PIHs in the transmission ecology and evolution of FMDV remains poorly understood.

Here, we investigate the persistence of FMDV within individual hosts in Vietnam. FMD is a highly contagious disease caused by FMDV in cloven‐hooved animals and is characterized by blisters and lesions in the oral cavity and on the feet. It is subject to strict international regulation due to its potential to cause significant economic impacts, which include production losses, culling of infected or at‐risk animals, and imposition of international trade restrictions [9]. FMDV is a positive‐sense, single‐stranded RNA virus, with seven distinct serotypes globally (O, A, C, SAT 1, SAT 2, SAT 3, and Asia 1) [10–13]. The FMDV genome has ~8000 nucleotides, consisting of a 5^′^ untranslated region and a single polyprotein that is processed after translation into various structural and nonstructural proteins. The P1 region is a ~2000 nucleotide portion of the FMDV genome that encodes the viral capsid proteins (VP1 through VP4). The P1 region and components therein are often used in epidemiological studies of FMDV due to their rapid rate of change, importance for classifying viral serotypes, and their interaction with the immune system [9]. Mutations in the P1 region have been associated with the carrier state and may play a role in viral persistence within the animal and in the host population, though these mutations are not consistent across studies [5, 10, 14–16].

Even though FMDV PIHs have been recognized since the 1950s [3, 17], only a few studies have explored the molecular changes in FMDV over time within PIHs under field conditions [18–20] or experimental settings [14, 21, 22]. Although difficult to demonstrate in field settings, PIHs harbor viral sub‐populations within which viral mutation occurs [18, 20]. Transmission from FMDV carriers has been shown to be epidemiologically important in African buffalo [23, 24], but natural transmission has not been demonstrated for cattle and water buffalo [3]. Nevertheless, if viruses from within carrier animals subsequently pass from carriers to other susceptible animals, within‐host evolution could have significant epidemiological ramifications [25].

In the current study, we use sequence data for serotypes A and O from a longitudinal field study in Vietnam from 2017 to 2019 to explore the within‐host evolution of FMDV in persistently infected animals with three primary goals: (1) to characterize specific sites of amino acid changes associated with persistent FMDV infections in individual cattle, (2) to determine the within‐host substitution rates across the VP1, P1 regions and whole genome of FMDV, and (3) to examine the potential epidemiological significance of FMDV carrier animals through examining phylogenetic relationships among sequences obtained from persistently (subclinically) infected animals and clinically infected animals. Our results suggest that certain serotype‐specific amino acid changes are associated with viruses isolated during the persistent phase of infection, that VP1 maintains a high mutation rate within PIHs, and that persistently infected cattle may play a role in FMDV population‐level maintenance and possibly serve as sources for disease outbreaks.

## 2. Results

In this study, 272 bovines (161 cattle, 69 buffalo, and 42 unspecified species) were sampled every 3–4 months during 2017–2019 across eight provinces in Vietnam, of which 69 had a FMDV‐positive oropharyngeal fluid (OPF) sample at the beginning of the study. Of these, we identified 38 animals (28 cattle, one buffalo, and nine unspecified bovines) that were potentially FMDV PIHs (i.e., animals with at least two FMDV‐positive OPF samples collected >21 days apart). None of these PIHs showed any clinical signs. Of these 38 possible PIHs, five cattle were identified as having distinct serial infections (sequential sequences of the P1 gene were >5% different, suggesting infection by a different sublineage) and 33 (28 of which were cattle) were identified as having persistent FMDV infections (sequential sequences were <2% different, interpreted as continued infection with the same virus). OPF samples from two animals (DT‐P037 and DT‐P115) yielded closely related consensus sequences at some successive timepoints and substantially divergent consensus sequences at others; these were therefore classified as showing both persistent and serial infection patterns at the consensus level (Figure 1).

**Figure 1:**
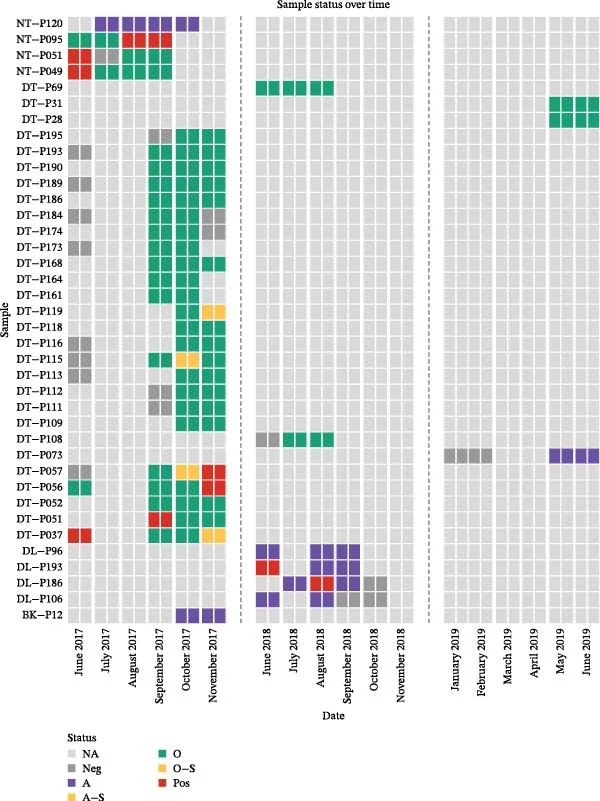
Summary of sampling history of potential carrier animals in a longitudinal sampling study for FMDV in Vietnam between 2017 and 2019. Information about the sampling timepoint is coded as colors: NA, no sample collected; Neg, OPF sample was FMDV‐negative; A, serotype A sequence obtained; O, serotype O sequence obtained; A–S and O–S, an additional sequence of the same serotype was obtained, but it was >5% different than the previous timepoint (i.e., a serial infection). In some cases (shown in red), samples were positive on qRT‐PCR positive results, but no sequence was obtained.

### 2.1. Evolutionary Patterns

The average within‐host substitution rate in PIHs varied by genome region, with VP1 having the highest estimated rate (0.081 mutations/nt site/yr.), followed by the coding DNA sequence (CDS) mutation rate of 0.069) and the rest of P1 (excluding VP1, 0.060). These rates are higher but still on the same order of magnitude as the substitution rates generated from a population‐level (122–26 serotype A and 96 serotype O cross‐sectional samples from clinical outbreaks and slaughterhouses in Vietnam) phylodynamic analysis, which estimated a substitution rate of 0.020 substitutions/nt site/yr. (95% HPD interval: 0.017–0.024) for serotype A and 0.015 substitutions/nt site/yr (95% HPD interval: 0.013–0.017) for serotype O.

### 2.2. Nonsynonymous Mutations in Samples From Carriers

For the P1 region, our dataset contained 10 and 35 paired sets of serotype A and serotype O samples from persistent infections, respectively (excluding serial infections) (Table 1). Each “sample pair” represents two time‐separated virus sequences from a single host. For serotype A persistent infections, 4 of 10 sample pairs had a nonsynonymous change in VP3 (site 438), and 5 of 10 sample pairs had a nonsynonymous mutation in VP1 (site 623, Table 1). There was evidence (significant *p*‐values and base factor support) of positive selection pressure acting at these amino acid sites and also several other sites, both at the within‐host and population levels. For serotype O persistent infections, 8 of 35 sample pairs had a nonsynonymous change in VP3 (site 378). Other amino acid sites where changes occurred in multiple persistent serotype O infections (four instances each) included VP1‐site 477, VP2‐site 218, and VP3‐site 376. Many of these amino acid sites were under positive selection pressures both at the within‐host and population levels (Table 1).

**Table 1:** P1 amino acid sites of interest from serotype A and O samples in carrier animals.

VP region	P1 amino acids	# Of time‐paired PIH sequences with aa change	SLAC P (dN/dS > 1)	FUBARP Bayes factor (α < β)	FLE (*p*‐value)	Positively selected in persistent infections	Positively selected in population at‐large
Serotype A (*N* = 10)							
3	478	0	0.999	63.741	0.030	—	✔
1	696	0	0.155	147.569	—	—	✔
1	623	5	0.000	1492788.2	—	✔	✔
1	665	2	0.004	4314.68	—	✔	✔
1	672	2	0.020	12929.2	—	✔	✔
2	219	2	0.069	52.892	<0.001	—	—
2	286	0	0.058	88.403	0.037	—	✔
3	438	4	0.004	3996.8	0.004	✔	✔
Serotype O (*N* = 35)							
3	426	1	0.118	187.219	0.045	—	✔
3	437	1	0.067	73.047	0.061	—	✔
3	477	4	0.001	487680.9	<0.001	✔	✔
3	498	3	0.036	4526.9	0.024	✔	✔
1	568	3	0.116	73.163	0.025	✔	✔
3	523	1	0.048	79.399	0.026	—	✔
1	569	0	0.016	6517.3	0.014	—	✔
1	720	3	0.622	3.008	—	—	—
1	721	3	0.821	1.261	—	—	—
2	124	3	0.571	12.993	0.029	—	—
2	159	2	0.004	125.72	0.021	—	✔
2	218	4	0.022	321.509	0.092	✔	✔
2	294	1	0.051	195.737	0.014	—	✔
3	376	4	0.250	2.544	0.005	✔	✔
3	378	8	0.078	2833.093	0.0026	✔	✔
3	379	0	0.003	4062.677	0.006	—	✔
4	43	3	0.958	0.05	0.0179	—	—

*Note:* Checkmarks indicate that the site was under positive selection in at least two out of three dN/dS methods.

Using the FMDV CDS, our dataset contained six and nine sets of paired samples for serotype A and O persistent infections, respectively. Each “sample pair” represents two time‐separated virus sequences from a single host. For serotype A, two of six had nonsynonymous mutations at amino acid site 420, 824, 1557, 1571, 2020, and 2072. All other changes were only present in the one sample pair. For serotype O, three of nine sample pairs had nonsynonymous mutations at amino acid site 921. Two of nine sample pairs had nonsynonymous mutations at amino acid sites 244, 360, 678, 922, 1200, 1710, 1850, and 2142.

To determine whether positively selected sites corresponded to previously characterized epitopes, we compared them to the serotype‐specific antigenic regions described by Cooke and Westover [26]. After converting P1 alignment coordinates to the full polyprotein numbering scheme using an alignment‐derived offset of 201 amino acids, several positively selected sites corresponded to previously described antigenic regions. In serotype O, positively selected positions 568 and 569 mapped to polyprotein positions 769 and 770 within the VP1 T‐helper epitope ETQIQRRQHTDVSFIMDRFV (positions 769–788) (Supporting Information 1: Table S1). In serotype A, positively selected position 696 mapped to polyprotein position 897 within overlapping VP1 B‐cell epitopes spanning positions 886–903 and 892–919. Serotype O VP3 position 379 was also mapped within the reported T‐helper epitope PFGHLTKLELPTDHH (positions 379–393). Several other positively selected sites did not overlap with previously described antigenic regions. Serotype A positions 665 and 672 mapped upstream of the VP1 B‐cell epitope cluster beginning at position 886, whereas the selected VP3 sites in serotype O mapped either upstream or downstream of the VP2 T‐helper epitope DVSLAAKHMSNTYLS (positions 603–617). Serotype O position 43 mapped within the VP4 T‐helper epitope SIINNYYMQQYQNSM (positions 240–254) but was not identified as positively selected in either analysis. The remaining positively selected sites occurred outside the antigenic regions reported by Cooke and Westover [26] (Supporting Information 1: Table S1).

#### 2.2.1. Discrete Trait Analysis (DTA)

Using Bayesian inference in BEAST, we performed a DTA to assess whether there was sequence‐based evidence that persistent infections resulted in onward transmission. In this analysis, we categorized sequences as being isolated from a clinical outbreak, PIH, or as being undetermined (Figure 2). “Undetermined” was used for subclinical samples for which the infection stage (persistent versus recent infection) could not be determined. We only categorized samples as coming from PIH if they were confirmed to be in the persistent phase of infection (i.e., evidence that they were FMDV‐positive for 21+ days). Thus, the initial timepoints for PIHs were categorized as “undetermined,” as we did not know for how long they had been FMDV‐positive. This categorization was to ensure that any inferred transmission event from PIHs occurred during the persistent phase of infection and not when the animal may have been in the early phase of infection. Samples from subclinical animals at slaughterhouses were also considered undetermined.

**Figure 2:**
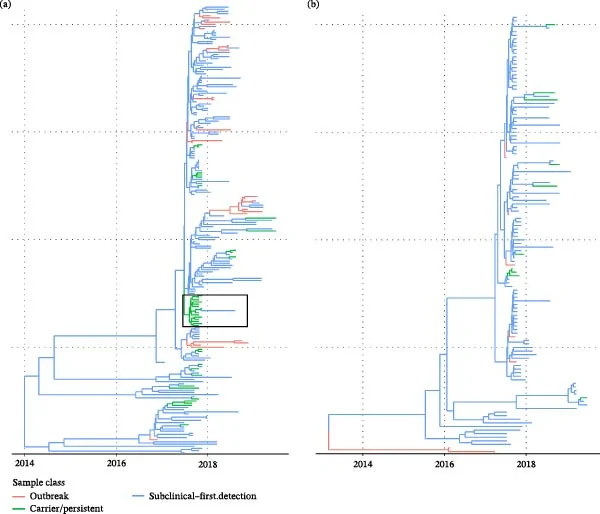
MCC phylogenetic trees obtained from BEAST tree reconstruction for serotypes O (a) and A (b). Highlighted by a black rectangle is an example of a clade where instances of onward transmission from PIHs were inferred.

Within serotype A samples, statistical support was only found for transmission from the undetermined infection state to persistent and outbreak samples (BF >100). Within serotype O samples, statistical support was found for transitions from undetermined to both outbreak and persistent infection samples, from outbreaks to undetermined, and most interestingly, from persistent infections to undetermined, all transitions with BF >3 and number of transitions ranging between 5 and 20 (Figure 3).

**Figure 3:**
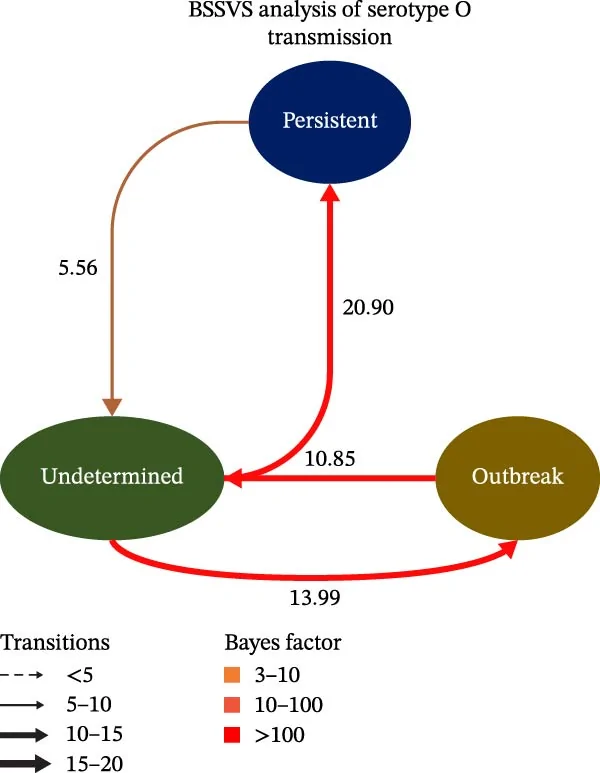
Summary of transition rates from discrete trait analysis of infection phase related to transmission for serotype O samples. Arrows are labeled with estimated number of transitions.

## 3. Discussion

The widespread controversy about the contagiousness of persistently infected cattle combined with zero tolerance for carriers in the determination of FMD‐free status and trade is an intrinsic conundrum in FMD control [3]. The observation of within‐host evolution of FMDV in carriers raises the possibility that persistently infected animals could act as reservoirs of genetic diversity and sources of new strains through onward transmission; however, experimental studies have failed to demonstrate natural transmission from persistently infected cattle, possibly because it is a very rare event. In the current study, we characterized within‐host evolution of FMDV in carriers under natural conditions in Vietnam and used a phylodynamic approach to provide statistical support for several instances where onward transmission appears to have occurred during the persistent infection phase of infection. In this endemic context, where subclinical circulation of multiple strains is recognized [27, 28]. Serial infection with genetically distinct strains of the same serotype was observed in 5 of 38 longitudinally sampled animals, indicating that sequential infection can occur under endemic field conditions. These findings suggest that (1) carriers may act as reservoirs of genetic diversification and potential strain emergence and (2) onward transmission can occur during the persistence phase.

### 3.1. Carriers May Serve as Reservoirs

Substitution rates within hosts were estimated from pairwise comparisons of longitudinal sequences obtained from the same PIH, whereas population‐level rates were estimated using Bayesian phylodynamic analyses in the BEAST program. Within‐host substitution rates (0.06–0.08 substitutions/site/year) were numerically higher than population‐level rates (0.01–0.02 substitutions/site/year); however, these estimates were derived using different analytical approaches, and formal statistical comparisons were not performed. Nonetheless, FMDV infections in persistently infected animals should not be viewed as a state of dormancy, and viral evolution continues throughout persistent infection. Although these within‐host estimates may reflect mutational processes operating within individual hosts, potential mechanisms driving these evolutionary processes should therefore be interpreted cautiously [29]. The within‐host substitution rates are not inconsistent with estimates published in other studies [14, 19, 30, 31] and also exhibited similar patterns in different regions of the genome (VP1 vs. P1 vs. CDS). Our finding that the VP1 genome structural protein region exhibited more rapid changes than the rest of the genome also aligned with previous research [14, 32–35]. The consistently higher rates of change observed in the VP1 region relative to other genomic regions at both population and within‐host levels suggest that immune pressure is a key driver of FMDV evolution across biological scales. This likely reflects the critical role of VP1 (including its G and H beta strands and the integrin‐binding region) in mediating immune recognition and receptor interactions [36–39]. The alignment of specific amino acid sites under positive selection pressure in the host population and the amino acid changes observed within persistently infected animals further suggests that selection pressures operating on within‐host microevolution in PIHs contribute to population‐level evolutionary patterns [40] and thus could have epidemiological consequences.

Specifically, from our dN/dS analyses, we observed that amino acid changes varied between serotypes, with serotype A exhibiting numerous alterations primarily in the VP1 region and fewer in VP3, while serotype O displayed a higher frequency of changes in VP3 (Table 1). Diversifying selection was evident in both serotypes in PIHs and population‐level samples, affecting critical antigenic sites in VP1, VP2, and VP3. Notably, in serotype A, the locations with frequent changes in PIHs also experienced diversifying selection in the broader host population, particularly in VP1 and VP3 (Table 1), suggesting similar selective pressures acting both within and between hosts. Moreover, serotype O’s consistent mutation patterns in the VP3 region across different phases of infection underscore its importance as a key area under the diversifying selection pressure. Previous studies have noted mutations in adjacent areas in FMDV persistently infected animals, though the specific implications of these changes have yet to be fully elucidated (Table 1) [1, 14, 16, 41].

The distribution of nonsynonymous changes across capsid proteins differed between serotypes, with serotype A showing more changes in VP1 and serotype O showing more changes in VP3. One possible explanation is that selective and structural constraints differ among capsid proteins and across FMDV serotypes. VP1 is prominently exposed on the virion surface and contains regions involved in receptor interaction and antibody recognition; thus, the predominance of changes in serotype A VP1 may reflect either greater tolerance for variation at exposed residues or stronger immune pressure on this protein. The higher frequency of changes in serotype O VP3 may indicate that, in the lineages examined, more immunologically accessible or structurally tolerant sites occurred in VP3. This is consistent with previous evidence that the conservation within antigenic regions varies among FMDV serotypes.

Diversifying selection pressure is not solely due to immune pressure but can also be influenced by other factors such as environmental heterogeneity and host‐pathogen interactions [42]. Moreover, it is likely that the host immune pressure during persistent infection is fundamentally different from that during acute infection. In persistent infection, the immune system may exert a more targeted and sustained pressure, leading to distinct evolutionary pathways compared to the rapid and broad immune response seen in acute infection [21, 43]. This could result in unique patterns of amino acid changes and diversifying selection pressures that are not observed during acute infection phases. This study adds further support that this region can be highly variable and may potentially have a role in persistent FMDV infection and in FMDV epidemiology.

The overlap of several selected sites with previously characterized antigenic regions suggests that immune pressure does contribute to FMDV evolution during persistent infection, although this pattern was not observed for most selected sites. In serotype O, positively selected sites fell within the VP1 T‐helper epitope ETQIQRRQHTDVSFIMDRFV and the VP3 T‐helper epitope PFGHLTKLELPTDHH, indicating that at least some substitutions under selection occurred in regions previously implicated in host immune recognition. In serotype A, one site mapped within overlapping VP1 B‐cell antigenic regions [26] (Supporting Information 1: Table S1). This is broadly consistent with the findings of Ramirez‐Carvajal et al. [20], who reported predominantly purifying selection during long‐term FMDV persistence and found no clear pattern of antigenic selection despite detecting a small number of positively selected codons. At the same time, most selected sites were located outside the antigenic regions described by Cooke and Westover [26], including several sites that showed strong support for positive selection in both the within‐host and population‐level analyses (Supporting Information 1: Table S1). This pattern is broadly consistent with the findings of Ramirez‐Carvajal et al. [20], who reported predominantly purifying selection during long‐term FMDV persistence and found no clear pattern of antigenic selection despite detecting a small number of positively selected codons. Taken together, these results suggest that while immune‐mediated selection may have contributed to variation at a subset of sites, sequence‐based analyses alone cannot determine whether these substitutions altered antigenicity or facilitated immune escape. Functional assays, including antibody binding and T‐cell recognition studies, will be needed to establish the biological significance of these changes.

### 3.2. Onward Transmission From Persistently Infected Animals

In the exploration for potential transmission from carriers to other animals using DTA, we observed suspected transmission from persistently infected animals (with serotype O), albeit at a low frequency. Our approach focused specifically on onward transmission associated with the phylogenetic clade nested within individual PIHs, wherein a cross‐sectional sample was found to be nested within the clade of sequences associated with the persistent phase of infection of a particular PIH. This provides field data supporting the possibility that onward transmission occurs from persistent infections in cattle. Natural transmission from carrier cattle and water buffalo to naïve animals has not been demonstrated [1, 8, 44], although transmission from persistently infected African buffalo is documented [24, 45].

Our findings and approach differ slightly with the study by Brito et al. [19], where phylodynamic analysis was used to suggest higher rates of onward transmission from carriers. However, in their study, all FMDV‐positive animals that lacked clinical signs were classified as “carriers,” without demonstrating the persistent nature of the infection. Determining what stage of infection a host may be in at the time of sampling is a major challenge in many field settings, especially since newly infected hosts do not always display clinical signs. Thus, this study may overemphasize the role of carrier animals in disease dynamics as the stage of infection during which transmission occurred cannot be determined. This contrasts with our data, where multiple sequences from different time points were available from single PIHs, and the initial timepoints were classified conservatively as an undetermined phase of infection. Using this conservative approach, we were able to uncover rare instances where onward transmission was inferred based on the within‐host diversity found in individual PIHs.

However, we acknowledge that we are not able to definitively show disease transmission events based on these data. This challenge can be addressed in future prospective studies. Moreover, the dates of samples reflect the date of collection, not infection; if the infection date was actually far before the collection date, this could change the topology of the time‐scaled tree and the observed nesting patterns. Finally, as is the case with numerous field studies, FMDV is under‐sampled, and thus phylodynamic inference may be based on only part of the transmission/evolutionary story of FMDV in this population. Future study designs could consider ways to ensure more holistic and inclusive sampling among the study populations.

### 3.3. Serial Infections

For many years, it has been recognized that a large proportion of FMDV‐infected animals will become carriers [18, 46–48]. Our data suggests that up to 13% of animals that are classed as carriers based on rt‐PCR results (i.e., those that remain persistently FMDV‐positive for ≥21 days) may actually be serially infected by distinct viruses or classified as both carriers and serially infected at different timepoints (5% in our dataset). However, because serial infection was inferred from the sequential recovery of substantially divergent consensus sequences, low‐frequency coinfection or changing dominance of viral lineages could not be excluded using the consensus‐based sequencing approach. In one of the only similar studies to date in Pakistan, Farooq et al. [18] demonstrated that among water buffalo carriers, about 62% were actually serially infected compared to only 38% being true carriers. These results suggest that caution is needed when defining carriers in the absence of sequence data as serial infections could inflate the true rate of persistently infected animals, particularly in regions with substantial FMDV diversity.

The high rate of serial infections also highlights the potential for hosts to be coinfected by multiple viral strains. Recombination has been recently recognized as a frequent evolutionary mechanism in FMDV, and carrier animals may be the source of new viral recombinants [3, 21, 25, 36, 49, 50]. Although recombination did not feature prominently in these data, it is plausible to consider a scenario where within‐host evolution and subsequent acquisition of a new infection in carriers could catalyze recombination events [36, 51].

### 3.4. Interpretive Context

In interpreting these findings, several considerations should be taken into account. Longitudinal sampling enabled persistent infection classification via temporal and genetic criteria, but precise initial infection timing remains uncertain, leaving some ambiguity in the transmission stages. Discrete 3–4 month sampling intervals likely missed intermediate variants and limited short‐term evolutionary resolution, though within‐host repeated sampling provided key insights. Like most field genomic studies, our dataset captures only a subset of circulating diversity, potentially affecting phylogenetic and transmission inferences. DTA offers statistical support for transmission patterns but cannot substitute for direct epidemiologic evidence. Finally, consensus sequences underrepresent within‐host diversity. These constraints are inherent to field molecular epidemiology but do not negate the demonstrated patterns of persistence and transmission.

Virus isolation (VI) prior to sequencing may also have contributed to the viral diversity observed in this study. Passage in cell culture can favor variants that are better adapted to growth under in vitro conditions and that may not fully represent the dominant viral population present in the host. Such selection can affect traits related to receptor usage, replication efficiency, and other aspects of viral fitness in culture. Because all sequences were generated from virus isolation supernatants, some mutations or changes in variant frequency could reflect adaptation during cell culture rather than evolutionary processes occurring in vivo. Nevertheless, longitudinal, outbreak, and slaughterhouse samples were processed using the same overall isolation and sequencing approach, making it unlikely that culture‐associated selection introduced systematic differences among these epidemiological groups. As direct sequencing data from the original OPF samples were not available, the relative contributions of culture‐associated and in vivo evolution could not be distinguished and should be considered when interpreting the observed genetic variation.

Because these analyses were based on consensus sequences, they cannot capture the full extent of within‐host viral diversity. Some of the substitutions observed between sampling points may reflect minority variants already present at the earlier time point, whereas others may represent newly emerged or fixed changes. In addition, the 3–4 month intervals between samples may have missed transient intermediate variants. Reanalyzing the original sequence reads with a validated minority‐variant method would be necessary to distinguish among these possibilities.

### 3.5. Key Findings

This study provides several key insights into the within‐host evolution of FMDV. Viral mutation rates within persistently infected animals are comparable to those measured at the epidemiological scale; within‐host evolution, combined with serial infection and viral recombination, may play a significant role in the ecology and evolution of FMDV. There are consistent amino acid sites under selection both at the population level and within persistently infected animals, indicating that selective pressures within and between hosts may be related, but the intensity of selection may vary slightly between serotypes. While serial infection in field settings and viral mutation are common occurrences, definitive evidence of onward transmission from persistently infected animals is lacking. The current work suggests that these mechanisms may occur concurrently and thereby contribute to the epidemiology of FMDV in endemic regions.

## 4. Methods

### 4.1. Sample Collection, Viral Isolation, and Sequencing

Samples were collected as part of a longitudinal study of 272 bovines (161 subclinical cattle, 69 buffalo, and 42 unspecified species) on farms in Vietnam between 2017 and 2019, as previously described [28]. Briefly, OPF samples were collected every 3–4 months on selected farms across eight provinces in Vietnam. Within each year, the same animals were sampled at a certain timepoint; however, sampled farms and animals differed between years. No recent FMD cases were reported in study herds based on farmer histories, though clinical status wasn’t independently verified at sampling, and subclinical/unreported infections remain possible. Vaccination dates weren’t systematically available across animals, so including status in the analysis risked misclassification and was avoided.

OPF samples were screened for FMDV using qRT‐PCR; then, positive samples were subjected to VI, followed by confirmation of FMDV in VI supernatant using qRT‐PCR [52]. Sequences were obtained from the VI supernatant by next‐generation sequencing (NGS) using the Illumina NextSeq platform (see [28] for details). Briefly, samples were sequenced using either RT‐PCR amplicons covering the P1 region [53] or using random and FMDV‐specific primers to obtain the near‐complete genome [54, 55]. Read quality filtering, trimming, de novo assembly, and assembly to previously published references of regionally endemic lineages were implemented in CLC Genomics Workbench v12 (Qiagen).

Additionally, viral sequences obtained from subclinical animals sampled at slaughterhouses (*n* = 90) and from clinical animals during outbreaks (*n* = 32) in the region between 2016 and 2019 were included in phylodynamic analyses. We refer to these samples as population‐at‐large samples. Samples were collected [28] and sequenced as described above. Sequences included in this study are available in GenBank (Supporting Information 2: Table S2).

We defined an infection in our dataset as any OPF sample that yielded infectious FMDV upon passage in LFBK‐αvβ6 cells (FMDV‐positive), as detailed in [56]. A FMDV‐positive OPF sample was classified as a persistent infection if its sampling date was ≥21 days after a previous positive sample from the same animal and if the P1 consensus sequence had a <2% pairwise nucleotide difference from the previous timepoint [43]. A FMDV‐positive OPF sample was classified as a serial infection (i.e., animal was infected with a different FMDV strain than in the previous timepoint) if the pairwise nucleotide difference between the P1 consensus sequences was >5%; this was interpreted as a new infection with likely a different viral sublineage. Therefore, while this classification indicates replacement by a substantially divergent dominant viral sequence, it does not exclude the prior presence of that lineage as a low‐frequency component of a mixed infection.

### 4.2. Preliminary Phylogenetic Analyses

In order to contextualize persistently and serially infected samples, we constructed maximum likelihood (ML) trees with all sequences from similar time periods and geographic locations (serotype A (*N* = 122) and O (*N* = 208). The two serotypes were analyzed independently. Sequences were aligned using MUSCLE [57] in Geneious Prime version 2022.0.2 (www.geneious.com). jModelTest version 2.1.10 [58] was used to determine the best substitution models for tree construction; both serotypes were best supported by the GTR + I + G substitution model. ML trees were subsequently constructed in Mega X with 1000 bootstraps [59]. P1 and CDS portions of all sequences were also screened using Recombination Detection Program 5 (RDP5) [60] in order to verify the absence of recombination events, which could impact downstream analyses.

#### 4.2.1. Substitution Rates Within‐Host

P1 (*N* = 47) and CDS (*N* = 15) sequences for serotypes A and O from persistently infected animals were compared in a pairwise, chronological process to determine the substitution rate of FMDV within PIHs. The number of differences between distinct sequential pairs of samples from within an individual animal at one timepoint compared to the next timepoint, that is, *t*_1_ versus *t*_2_ (*t*_*n*_ *–t*_*n* + 1_), was divided by the total number of residues in a sequence (yielding the value of number of substitutions per site). This value was then corrected for time between samples to generate a substitution rate (number of substitutions per site per year (substitutions/site/yr.). Substitution rates between VP1versus VP2–VP4 were compared to determine differences in evolutionary rates across these genomic regions within persistent infections; CDS portions were used to estimate substitution rates across the entire coding region of FMDV. Analyses were done separately for the two serotypes.

#### 4.2.2. Nonsynonymous Amino Acid Changes in Persistently and Serially Infected Animals

P1 sequences were translated in Geneious Prime version 2022.0.2. Changes were identified by comparing translated consensus sequences from sequential samples. Reported amino acid differences therefore reflect consensus‐level substitutions and do not account for within‐sample minority variants. Amino acid sequences were then analyzed for within‐host amino acid changes in a pairwise, chronological process. Additionally, dN/dS analysis was run on all sequences (excluding possible recombinants) separately for each serotype (serotype A: *N* = 121 sequences, serotype O: *N* = 169 sequences) using the single line alignment with context (SLAC) (*p*‐value ≤ 0.05), fast, unconstrained Bayesian AppRoximation (FUBAR) (posterior probability ≥0.9), and fixed effects likelihood (FEL) (*p*‐value ≤ 0.05) methods to detect positions under selective pressure at the population level [61–63]. These sequence sets contained the same samples as those used for the ML trees, except that only the earliest sequence was selected from each persistently infected animal in order to prevent over‐representation of sequences from single hosts. An identical analysis was also performed for CDS sequences from persistent infections to determine regions of change across the entire protein‐coding region. Analyses were done separately for the two serotypes.

### 4.3. Phylodynamic Analysis

The ML trees were uploaded into TempEst version 1.5.3 [64] to evaluate the temporal signal in the evolution of the viral sequences. A correlation coefficient (*R* ^2^) of >0.6 for both serotypes was observed, which is appropriate for further phylodynamic analysis of the data. A and O sequences were analyzed in BEAST v1.10.4 [65], using DTAs with Bayesian stochastic search variable selection (BSSVS) to evaluate evidence of transmission from persistently infected animals. To address uncertainties in how to classify samples into discrete traits representing persistent infections (Table 1), we created three discrete traits: outbreak (sequences collected during clinical outbreaks), persistent infections (<2% genetic distance from initial sequence in longitudinally sampled animals), and undetermined (Table 2). The first sequence from a longitudinally sampled animal was classified as undetermined as persistent infection status of the animal cannot be concluded without data from an earlier sampling point. Serial infections and cross‐sectional samples from subclinical animals were also classified as undetermined. All BEAST‐DTA analyses were conducted using the GTR + I + G substitution model, an uncorrelated relaxed clock with a lognormal distribution, and a Bayesian Skygrid coalescent model. The analyses involved 322 million MCMC iterations with a 32 million iteration burn‐in for serotype A and 381 million MCMC iterations with a 38 million iteration burn‐in for serotype O.

**Table 2:** Definitions of traits used in the discrete trait phylodynamic analysis.

Sample type	Discrete trait (outbreak, undetermined, and persistent
Slaughterhouse (cross‐sectional sampling from subclinical animals)	Undetermined
Clinical outbreak (cross‐sectional sampling from clinical animals)	Outbreak
Longitudinal sample: subclinical first detection with no subsequent positive samples	Undetermined
Longitudinal sample: subclinical first detection with subsequent positive samples	Undetermined
Longitudinal sample: subclinical second detection (<2% dissimilarity from pervious sample)	Persistent infection
Longitudinal sample: subclinical detection of new viral sublineage (>5% dissimilarity from previous sample	Undetermined

## Author Contributions

Dennis N. Makau and J. Trevor Vannatta conducted the data analyses, interpreted the results, and drafted the manuscript. Miranda Bertram and Ian Fish processed the samples and performed the bioinformatic analyses. Jonathan Arzt and Kimberly VanderWaal conceived and designed the study.

## Funding

This project was supported by the USDA Agricultural Research Service (Grant 58‐8064‐2‐006).

## Disclosure

All authors contributed to the review and revision of the manuscript, approved the final version, and agree to be accountable for all aspects of the work.

## Ethics Statement

Ethical review and approval was not required for the animal study because the work described herein was performed by the federal staff of the Department of Animal Health, Ministry of Agriculture and Rural Development, Government of Vietnam. The work occurred, and the animals were maintained within facilities that were owned, maintained, or overseen by these divisions of the federal governments; thus, no permits or approvals were required. All cases described herein occurred spontaneously in domestic cattle or buffalo with no experimentation, inoculation, or treatment of live animals.

## Conflicts of Interest

The authors declare no conflicts of interest.

## Supporting Information

Additional supporting information can be found online in the Supporting Information section.

## Supporting information


**Supporting Information 1** Table S1: Mapping of positively selected sites to previously described FMDV antigenic regions reported by Cooke and Westover (2008) (1). P1 alignment coordinates were converted to full‐polyprotein coordinates using an alignment‐derived offset of 201 amino acids. For each selected site, the table reports the corresponding polyprotein position, selection statistics (SLAC and FUBAR), support for selection in within‐host and population‐level analyses, overlap with previously described epitopes, and the distance to the nearest known antigenic region where applicable.

**Supporting Information 2** Table S2: List and metadata of FMDV sequences obtained from GenBank for a study on within‐host evolution and carrier status

## Data Availability

VP1 consensus sequences are deposited in GenBank (Supporting Information 2: Table S2). Associated metadata are available from the corresponding author upon reasonable request, subject to ongoing analyses and institutional data policies.
