# Supplementary material for: Within‐Host Evolution of Foot‐and‐Mouth Disease Virus From Persistently Infected Cattle and Buffaloes in Vietnam
Source: Transbound Emerg Dis. 2026 Oct 23;2026:3373628. doi: 10.1155/tbed/3373628 (PMC13692833; doi:10.1155/tbed/3373628)
Supplement: Supplementary file 1 [file TBED-2026-3373628-s001.docx]

**Supplementary table s2: List and metadata of FMDV sequences obtained from GenBank for a study on within-host evolution and carrier status**

| VP1 accession | Host | Sample Type | Date | Lineage |
| --- | --- | --- | --- | --- |
| KT153077 | Cattle | outbreak | 9-Jan-09 | A Asia/Sea-97 |
| KT153078 | Pig | outbreak | 16-Oct-12 | A Asia/Sea-97 |
| KT153078 | Pig | outbreak | 16-Oct-12 | A Asia/Sea-97 |
| KT153079 | Cattle | outbreak | 9-Oct-13 | A Asia/Sea-97 |
| KT153079 | Cattle | outbreak | 9-Oct-13 | A Asia/Sea-97 |
| KT153080 | Cattle | outbreak | 15-Jan-10 | A Asia/Sea-97 |
| KT153081 | Buffalo | outbreak | 16-Feb-09 | A Asia/Sea-97 |
| KT153082 | Pig | outbreak | 17-Feb-09 | A Asia/Sea-97 |
| KT153083 | Buffalo | outbreak | 19-Feb-09 | A Asia/Sea-97 |
| KT153084 | Buffalo | outbreak | 17-Mar-09 | A Asia/Sea-97 |
| KT153176 | Cattle | outbreak | 1-Oct-10 | O ME-SA/PanAsia |
| KT153130 | Cattle | outbreak | 21-Dec-10 | O ME-SA/PanAsia |
| KT153132 | Cattle | outbreak | 22-Dec-10 | O ME-SA/PanAsia |
| KT153129 | Buffalo | outbreak | 26-Dec-10 | O ME-SA/PanAsia |
| KT153085 | Buffalo | outbreak | 1-Jan-11 | O ME-SA/PanAsia |
| KT153087 | Buffalo | outbreak | 1-Jan-11 | O ME-SA/PanAsia |
| KT153088 | Cattle | outbreak | 5-Jan-11 | O ME-SA/PanAsia |
| KT153094 | Cattle | outbreak | 5-Jan-11 | O ME-SA/PanAsia |
| KT153110 | Cattle | outbreak | 11-Jan-11 | O ME-SA/PanAsia |
| KT153113 | Cattle | outbreak | 11-Jan-11 | O ME-SA/PanAsia |
| KT153115 | Buffalo | outbreak | 14-Jan-11 | O ME-SA/PanAsia |
| KT153127 | Cattle | outbreak | 14-Jan-11 | O ME-SA/PanAsia |
| KT153140 | Buffalo | outbreak | 19-Jan-11 | O ME-SA/PanAsia |
| KT153141 | Pig | outbreak | 21-Jan-11 | O ME-SA/PanAsia |
| KT153162 | Buffalo | outbreak | 26-Jan-11 | O ME-SA/PanAsia |
| KT153163 | Buffalo | outbreak | 26-Jan-11 | O ME-SA/PanAsia |
| KT153164 | Pig | outbreak | 30-Jan-11 | O ME-SA/PanAsia |
| KT153170 | Pig | outbreak | 9-Feb-11 | O ME-SA/PanAsia |
| KT153174 | Pig | outbreak | 11-Feb-11 | O ME-SA/PanAsia |
| KT153175 | Buffalo | outbreak | 14-Feb-11 | O ME-SA/PanAsia |
| KT153177 | Pig | outbreak | 15-Feb-11 | O ME-SA/PanAsia |
| KT153178 | Pig | outbreak | 15-Feb-11 | O ME-SA/PanAsia |
| KT153179 | Buffalo | outbreak | 16-Feb-11 | O ME-SA/PanAsia |
| KT153106 | Pig | outbreak | 17-Feb-11 | O ME-SA/PanAsia |
| KT153182 | Buffalo | outbreak | 17-Feb-11 | O ME-SA/PanAsia |
| KT153108 | Pig | outbreak | 22-Feb-11 | O ME-SA/PanAsia |
| KT153099 | Cattle | outbreak | 1-Mar-11 | O ME-SA/PanAsia |
| KT153103 | Buffalo | outbreak | 2-Mar-11 | O ME-SA/PanAsia |
| KT153107 | Cattle | outbreak | 4-Apr-11 | O ME-SA/PanAsia |
| KT153117 | Pig | outbreak | 31-Oct-11 | O ME-SA/PanAsia |
| KT153118 | Pig | outbreak | 31-Oct-11 | O ME-SA/PanAsia |
| KT153181 | Pig | outbreak | 16-Jan-12 | O ME-SA/PanAsia |
| KT153119 | Pig | outbreak | 14-Mar-12 | O ME-SA/PanAsia |
| KT153123 | Pig | outbreak | 14-Mar-12 | O ME-SA/PanAsia |
| KT153124 | Pig | outbreak | 14-Mar-12 | O ME-SA/PanAsia |
| KT153125 | Pig | outbreak | 14-Mar-12 | O ME-SA/PanAsia |
| KT153134 | Pig | outbreak | 14-Mar-12 | O ME-SA/PanAsia |
| KT153138 | Pig | outbreak | 24-Mar-12 | O ME-SA/PanAsia |
| KT153137 | Cattle | outbreak | 11-Apr-12 | O ME-SA/PanAsia |
| KT153139 | Cattle | outbreak | 11-Apr-12 | O ME-SA/PanAsia |
| KT153096 | Pig | outbreak | 5-Nov-12 | O ME-SA/PanAsia |
| KT153171 | Pig | outbreak | 17-May-13 | O ME-SA/PanAsia |
| KT153089 | Cattle | outbreak | 6-Aug-13 | O ME-SA/PanAsia |
| KT153091 | Cattle | outbreak | 13-Aug-13 | O ME-SA/PanAsia |
| KT153097 | Pig | outbreak | 9-Oct-13 | O ME-SA/PanAsia |
| KT153105 | Cattle | outbreak | 30-Oct-13 | O ME-SA/PanAsia |
| KT153122 | Cattle | probang | 23-Apr-12 | O ME-SA/PanAsia |
| KT153142 | Cattle | probang | 13-Apr-12 | O ME-SA/PanAsia |
| KT153143 | Cattle | probang | 17-May-12 | O ME-SA/PanAsia |
| KT153155 | Cattle | probang | 4-Feb-13 | O ME-SA/PanAsia |
| KT153145 | Cattle | probang | 3-Jul-12 | O ME-SA/PanAsia |
| KT153150 | Cattle | probang | 4-Sep-12 | O ME-SA/PanAsia |
| KT153158 | Cattle | probang | 17-Jun-13 | O ME-SA/PanAsia |
| KT153152 | Cattle | probang | 5-Oct-12 | O ME-SA/PanAsia |
| KT153144 | Cattle | probang | 7-Jun-12 | O ME-SA/PanAsia |
| KT153146 | Cattle | probang | 7-Jul-12 | O ME-SA/PanAsia |
| KT153151 | Cattle | probang | 4-Sep-12 | O ME-SA/PanAsia |
| KT153153 | Cattle | probang | 5-Oct-12 | O ME-SA/PanAsia |
| KT153154 | Cattle | probang | 3-Jan-13 | O ME-SA/PanAsia |
| KT153156 | Cattle | probang | 4-Feb-13 | O ME-SA/PanAsia |
| KT153157 | Cattle | probang | 17-Jun-13 | O ME-SA/PanAsia |
| KT153098 | Buffalo | probang | 9-May-12 | O ME-SA/PanAsia |
| KT153161 | Buffalo | probang | 5-Nov-12 | O ME-SA/PanAsia |
| KT153116 | Buffalo | probang | 23-May-12 | O ME-SA/PanAsia |
| KT153159 | Buffalo | probang | 10-Nov-12 | O ME-SA/PanAsia |
| KT153104 | Buffalo | probang | 23-May-12 | O ME-SA/PanAsia |
| KT153128 | Cattle | probang | 23-Apr-12 | O ME-SA/PanAsia |
| KT153168 | Cattle | probang | 23-May-12 | O ME-SA/PanAsia |
| KT153131 | Cattle | probang | 7-Jul-12 | O ME-SA/PanAsia |
| KT153169 | Cattle | probang | 13-Oct-12 | O ME-SA/PanAsia |
| KT153166 | Cattle | probang | 11-Nov-12 | O ME-SA/PanAsia |
| KT153111 | Cattle | probang | 7-Jul-12 | O ME-SA/PanAsia |
| KT153147 |  | probang |  | O ME-SA/PanAsia |
