# Supplementary material for: Within‐Host Evolution of Foot‐and‐Mouth Disease Virus From Persistently Infected Cattle and Buffaloes in Vietnam
Source: Transbound Emerg Dis. 2026 Oct 23;2026:3373628. doi: 10.1155/tbed/3373628 (PMC13692833; doi:10.1155/tbed/3373628)
Supplement: Supplementary file 2 [file TBED-2026-3373628-s002.docx]

| **Serotype** | **VP Region** | **P1 Position** | **Polyprotein Position** | **PIH Selection** | **Population Selection** | **Nearest/Overlapping Epitope** | **Epitope Coordinates** | **Relationship** |
| --- | --- | --- | --- | --- | --- | --- | --- | --- |
| A | VP1 | 478 | 679 | No | Yes | None identified | — | Outside known epitopes |
| A | VP1 | 623 | 824 | Yes | Yes | VP1 B-cell cluster | 886-903 / 892-919 | 62 aa upstream |
| A | VP1 | 665 | 866 | Yes | Yes | VP1 B-cell cluster | 886-903 / 892-919 | 20 aa upstream |
| A | VP1 | 672 | 873 | Yes | Yes | VP1 B-cell cluster | 886-903 / 892-919 | 13 aa upstream |
| A | VP1 | 696 | 897 | No | Yes | YNRNAVPNLRGDLQV / SRRGDLGLSLATRVATQLPAS | 886-903 / 892-919 | Within epitope |
| A | VP2 | 219 | 420 | No | No | None identified | — | Outside known epitopes |
| A | VP3 | 286 | 487 | No | Yes | None identified | — | Outside known epitopes |
| A | VP3 | 438 | 639 | Yes | Yes | DVSLAAKHMSNTYLS | 603-617 | 22 aa downstream |
| O | VP1 | 426 | 627 | No | Yes | DVSLAAKHMSNTYLS | 603-617 | 10 aa downstream |
| O | VP1 | 437 | 638 | No | Yes | DVSLAAKHMSNTYLS | 603-617 | 21 aa downstream |
| O | VP1 | 477 | 678 | Yes | Yes | None identified | — | Outside known epitopes |
| O | VP1 | 498 | 699 | Yes | Yes | None identified | — | Outside known epitopes |
| O | VP1 | 523 | 724 | No | Yes | ETQIQRRQHTDVSFIMDRFV | 769-788 | 45 aa upstream |
| O | VP1 | 568 | 769 | Yes | Yes | ETQIQRRQHTDVSFIMDRFV | 769-788 | Within epitope |
| O | VP1 | 569 | 770 | No | Yes | ETQIQRRQHTDVSFIMDRFV | 769-788 | Within epitope |
| O | VP1 | 720 | 921 | No | No | RTLPTSFNY | 914-922 | Within epitope |
| O | VP1 | 721 | 922 | No | No | RTLPTSFNY | 914-922 | Within epitope |
| O | VP2 | 124 | 325 | No | No | None identified | — | Outside known epitopes |
| O | VP2 | 159 | 360 | No | Yes | PFGHLTKLELPTDHH | 379-393 | 19 aa upstream |
| O | VP2 | 218 | 419 | Yes | Yes | None identified | — | Outside known epitopes |
| O | VP3 | 294 | 495 | No | Yes | None identified | — | Outside known epitopes |
| O | VP3 | 376 | 577 | Yes | Yes | DVSLAAKHMSNTYLS | 603-617 | 26 aa upstream |
| O | VP3 | 378 | 579 | Yes | Yes | DVSLAAKHMSNTYLS | 603-617 | 24 aa upstream |
| O | VP3 | 379 | 580* | No | Yes | PFGHLTKLELPTDHH† | 379-393 | Within VP3 epitope† |
| O | VP4 | 43 | 244 | No | No | SIINNYYMQQYQNSM | 240-254 | Within epitope |

Supplementary Table S1. Mapping of positively selected sites to previously described FMDV antigenic regions reported by Cooke and Westover (2008)(1). P1 alignment coordinates were converted to full-polyprotein coordinates using an alignment-derived offset of 201 amino acids. For each selected site, the table reports the corresponding polyprotein position, selection statistics (SLAC and FUBAR), support for selection in within-host and population-level analyses, overlap with previously described epitopes, and the distance to the nearest known antigenic region where applicable.

**Footnotes**

* Polyprotein position 580 is obtained using the +201 offset. However, VP3 position 379 corresponds directly to the VP3 T-helper epitope PFGHLTKLELPTDHH described by Cooke & Westover because that epitope was reported using VP3 residue numbering rather than the renumbered P1 coordinate system.

† This is the strongest direct overlap between a selected site and a previously reported epitope in your dataset (SLAC P = 0.003; FUBAR BF = 4062.68).

1.	Cooke JN, Westover KM. Serotype-specific differences in antigenic regions of foot-and-mouth disease virus (FMDV): a comprehensive statistical analysis. Infect Genet Evol [Internet]. 2008 Dec [cited 2026 Aug 4];8(6):855–63. Available from: https://pubmed.ncbi.nlm.nih.gov/18790086/
